# Cerebrospinal fluid cell-free DNA as a liquid biopsy tool for detecting and monitoring genomic alterations in thalamic colorectal cancer metastases

**DOI:** 10.1093/noajnl/vdaf178

**Published:** 2025-10-14

**Authors:** Ali Gharibi Loron, Yooree Ha, Cecile Riviere-Cazaux, Xiaohong Wang, Arthur E Warrington, Terry C Burns

**Affiliations:** Department of Neurological Surgery, Mayo Clinic, Rochester, Minnesota, USA; Department of Neurological Surgery, Mayo Clinic, Rochester, Minnesota, USA; Department of Neurological Surgery, Mayo Clinic, Rochester, Minnesota, USA; Predicine, Inc., Hayward, California, USA; Department of Neurological Surgery, Mayo Clinic, Rochester, Minnesota, USA; Department of Neurological Surgery, Mayo Clinic, Rochester, Minnesota, USA

**Keywords:** brain tumor, cell-free DNA, liquid biopsy, next-generation sequencing

## Abstract

**Background:**

Cerebrospinal fluid cell-free DNA (cfDNA) can detect and monitor leptomeningeal disease but has not been previously used to monitor parenchymal lesions.

**Methods:**

Herein, we report our initial experience with CSF cfDNA monitoring for 2 patients with colorectal cancer (CRC) metastases to the thalamus, causing obstructive hydrocephalus.

**Results:**

CSF samples were obtained during ventriculoperitoneal shunt placement, demonstrating high levels of cfDNA in both cases. Several genomic alterations detected in the cfDNA sequencing matched those in the tumor tissue biopsy. Follow-up CSF evaluations after subsequent therapy were used to help adjudicate pseudo-progression versus true progression.

**Conclusions:**

Neither patient developed leptomeningeal disease, demonstrating CSF’s utility in evaluating solitary brain metastases in direct contact with a CSF compartment.

**Key Points**

- Cerebrospinal fluid (CSF) cell-free DNA (cfDNA) can assess brain metastases in direct contact with a CSF compartment.
- Next-Generation Sequencing of CSF cfDNA from metastases on the ependymal surface can detect molecular alterations consistent with tumor tissue.

**Importance of the Study**

Cerebrospinal fluid cell-free DNA (CSF cfDNA) analysis via Next-Generation Sequencing shows promise in detecting molecular alterations in brain metastases with ependymal contact. This study highlights the potential of CSF cfDNA as a liquid biopsy, especially in cases requiring CSF diversion. CSF obtained during neurosurgical procedures can undergo molecular testing, aiding in diagnosing and monitoring brain cancers. Importantly, tumor-derived cfDNA in CSF does not necessarily indicate leptomeningeal disease but may reflect metastatic tumors with CSF contact. This underscores CSF cfDNA’s role in central nervous system disease monitoring and its potential to complement or replace invasive biopsies.

Cerebrospinal fluid (CSF) analysis shows early promise as a source of biomarkers for detecting and monitoring central nervous system (CNS) tumors.^1,2^ Higher CSF cell-free DNA (cfDNA) levels have been reported in patients with CNS metastatic leptomeningeal disease (LMD).^3^ Tumor-derived CSF cfDNA is also detectable in about half of patients with infiltrative gliomas, even without the presentation of LMD,^4^ and has demonstrated potential for longitudinal disease monitoring.^5^ Unsurprisingly, higher levels of CSF cfDNA have been observed in patients whose gliomas come directly in contact with the ventricular or pial surface.^6^

Occasional metastatic tumors may abut the lateral ventricle in the absence of LMD. To our knowledge, only 1 study has previously reported that metastatic tumors contacting the lateral ventricle could be detected via CSF cfDNA (*n* = 2).^3^ Colorectal cancer (CRC) metastases to the brain are relatively uncommon, occurring in approximately 3% of CRC patients.^7^ Although a brain biopsy can be considered in uncertain cases, it carries a risk that could be averted with alternate methods. CSF diversion may be indicated in some cases of metastatic disease, including LMD with communicating hydrocephalus or obstructive hydrocephalus. In these cases, cranial CSF may be accessible via a ventriculoperitoneal (VP) shunt.

Herein, we present 2 cases of CRC with thalamic metastases requiring CSF diversion. Genomic testing was performed on the metastatic brain lesion or the colorectal mass. We asked whether the diagnosis could have been achieved based on genomic analysis of CSF obtained at the time of ventriculoperitoneal shunt placement without tissue biopsy and whether CSF could have potential relevance for longitudinal monitoring.

## Case Reports

### Case 1

**Figure 1.**
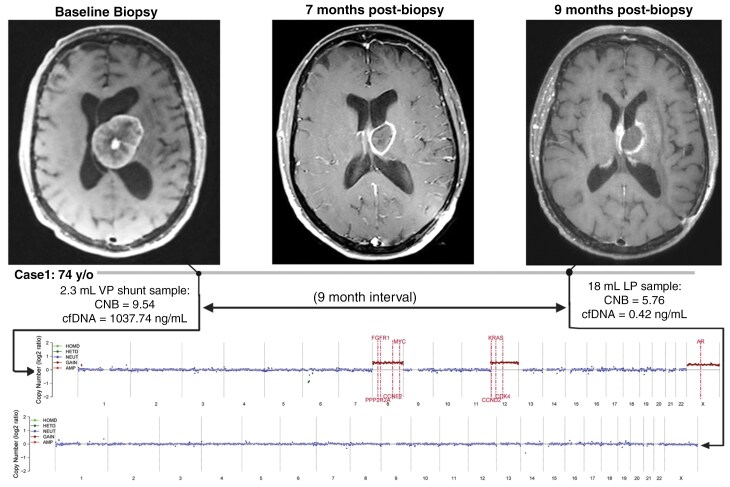
T1-weighted post-gadolinium MR images of Case 1. Left: Preoperative image. Middle: Restaging image while the patient had stable disease. Right: Post-radiation therapy images while the patient becomes symptomatic, 9 months after tumor biopsy. Below, the chromosomal microarray analysis of the patient’s samples is shown. CNB, copy number burden.

A 74-year-old female presented with several weeks of increasing confusion, mild aphasia, gait abnormalities, weakness, and falls. The patient had a known MMR-deficient adenocarcinoma of the proximal transverse colon with loss of PMS2 and MLH1 (Stage IIIc: pT4a, N2b, M0), for which she had undergone chemotherapy with adjuvant folinic acid, fluorouracil, and oxaliplatin (FOLFOX). Imaging at the presentation revealed a 4.5-cm^2^ enhancing mass centered in the left thalamus with substantial intraventricular extension and early obstructive hydrocephalus (Figure 1). Given the patient’s known colorectal adenocarcinoma, a brain metastasis was felt to be the most likely diagnosis. However, oncology requested confirmatory tissue. The patient underwent left-sided parieto-occipital ventriculoperitoneal (VP) shunt placement, and a stereotactic needle biopsy was performed under the same anesthesia via a different trajectory, avoiding the ventricle. Pathologic findings confirmed metastatic colorectal adenocarcinoma. Genomic testing of the thalamic lesion via Next-Generation Sequencing (NGS) redemonstrated molecular features of the primary disease, including BRAF mutation and loss of MLH1 NGS.^8^ Immunohistochemistry and methylation testing also confirmed the patient’s primary disease.

Per routine, the patient consented to our institutional neuro-oncology biorepository before surgery, and the cerebrospinal fluid (CSF) acquired from VP shunt placement was biobanked. At the time of concern for tumor recurrence based on MRI, CSF was retrospectively utilized for evaluation of tumor-associated cfDNA via the PredicineCARE panel. As previously detailed, the PredicineCARE panel analyzes 152 cancer-related genes across 587Kb genome regions.^9^ The CSF cfDNA concentration was 1037.74 ng/mL, which was a high concentration according to our team experience. Tumor-associated mutations were identified, including BRAF, with high microsatellite instability (MSI) and without loss of MLH. Genomic alterations were assessed via low-pass whole genome sequencing, revealing gains or amplifications in CDK4, FGFR1, PIK3CA, and KRAS, among other alterations, as detailed in Table 1. Prior testing of the colon tissue demonstrated numerous mutations in genes, including PIK3CA, BRAF, and frameshift mutations, including but not limited to those in NF1, as observed in CSF. The patient subsequently underwent radiation, followed by pembrolizumab, consistent with current recommendations for patients with known MSI.

**Table 1.** Next-Generation Sequencing Results From CSF in Case 1

Gene	Alteration	Allele freq./copy number
CDK4	Amplification	3.43
**PIK3CA**	Q546E	1.15%
**BRAF**	V600E	45.96%
FGFR1	Amplification	2.92
BRCA1	K654Sfs47	0.70%
BRCA1	Q19	0.26%
**NF1**	F358Lfs18	5.61%
**NF1**	R816	0.35%
TP53	R337C	0.30%
TP53	R175H	0.22%
MYC	Amplification	2.95
CCND2	Amplification	2.86
EGFR	G863R	0.46%
ERBB3	Amplification	3.26
PTPN11	Amplification	3.07
KDM6A	Amplification	2.83
MDM2	Amplification	2.65
KRAS	Amplification	2.47

Mutations detected in brain biopsy tissue based on a selected panel of genes are highlighted in bold. Microsatellite instability is highly detected.

Nine months later, the MRI showed concern for possible progression versus radiation necrosis. She was started on steroids for presumed radiation necrosis. The patient underwent a lumbar puncture to determine the presence and amount of viable tumor that is exophytic into the lateral ventricle, which was negative for malignancy. Lumbar puncture revealed 0.42 ng/mL cfDNA. This was insufficient to detect any specific alterations. However, the Predicine score assay uses a low-pass sequencing algorithm, showing a copy number burden (CNB) abnormality score of 5.76, down from 9.54 for the original CSF. Visual inspection of the whole genome plot did not reveal evidence of the original tumor-associated copy number variations (Figure 1). Restaging MRI imaging locally had not shown active cancer. She was initially treated with steroids and bevacizumab. Steroids were tapered off due to lack of improvement and concern for steroid myopathy. She was unable to walk with a walker and was using a wheelchair. The patient continued to experience neurological decline and weakness and was transferred to a hospice care facility. She passed away 26 months after her brain metastasis.

### Case 2

**Figure 2.**
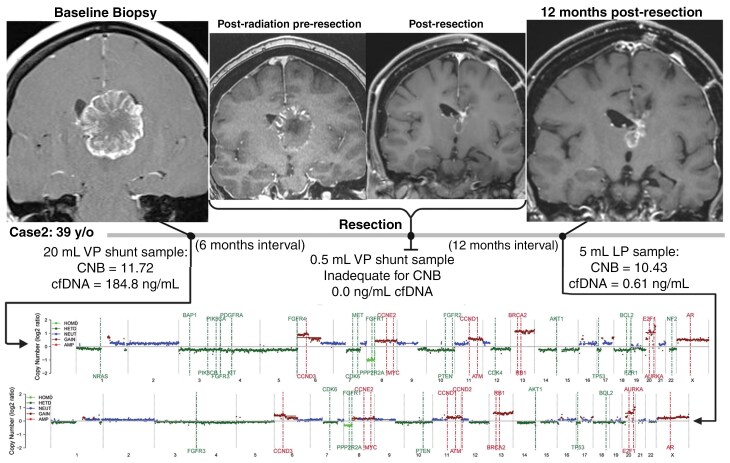
T1-weighted post-gadolinium MR images of Case 2. Left: Preoperative image for shunt placement. Middle: Pre- and postoperative images for tumor resection after 6 months. Right: Post-radiation therapy image, 12 months after tumor resection. Below, the chromosomal microarray analysis of the patient’s samples is shown. CNB, copy number burden.

A 39-year-old female with a known history of metastatic colon adenocarcinoma presented with a 2-week history of worsening daily headaches with nausea and vomiting. Imaging at presentation revealed a 4.3 cm^2^ heterogeneously enhancing mass centered in the left thalamus with asymmetric enlargement of the left lateral ventricle (Figure 2). The patient underwent VP shunt placement for her hydrocephalus. She had consented to the neuro-oncology biorepository, and her CSF was banked. A sample of her banked CSF was subsequently sent for cfDNA analyses on the PredicineCARE panel. CSF analysis revealed 184.80 ng/mL of cfDNA. The patient’s primary adenocarcinoma had a known KRAS G12D mutation and was negative for microsatellite instability. NGS of the CSF cfDNA via PredicineCARE revealed the same G12D KRAS mutation, in addition to TP53 and APC mutations, the combination of which is the most common genetic alteration in colorectal cancer.^10^ Additional alterations included CCND1, MYC, and AR amplifications and deletions in PTEN, APC, and TP53. No microsatellite instability was detected in the cfDNA, which is consistent with the findings of primary colorectal tumor tissue. The patient then underwent fractionated radiation. Six months later, the patient underwent resection of the tumor on a prospective clinical trial for resection of pre-recurrent disease (NCT04810871). At the time of surgery, CSF sampling from the shunt was attempted, but only 0.5 mL was obtained due to collapsed ventricles. This was sent to Predicine but contained 0.0 ng cfDNA, precluding further testing.

Eighteen months later, a lumbar puncture was performed due to equivocal concern for recurrence on brain MRI. Imaging revealed increased thickness of enhancement in the left thalamic lesion. A lumbar CSF sample containing 0.61 ng/mL cfDNA (compared to 184.80 ng/mL originally obtained from the shunt before cytoreductive therapies). Although >5 ng of cfDNA is preferred for the PredicineCARE NGS assay, it was performed with the ~3 ng cfDNA available, revealing persistent MYC, P53, and KRAS gene alterations and 7 new cancer-related gene mutations (Table 2). CNB score was 11.72, then 10.43 (Figure 2). Unfortunately, the patient experienced a progression of systemic metastatic disease involving multiple organs and was subsequently transferred to hospice care.

**Table 2.** Next-Generation Sequencing results From Case 2. KRAS, MYC, and TP53 Gene Alteration Are Detected on Follow-Up

Gene (sample at biopsy)	Gene (sample at 18-month follow-up)	Alteration	Allele freq./copy number
TP53		I254N	80.84%
APC		E941	78.35%
**KRAS**	**KRAS**	G12D	57.30% → 2.12
CCND1		Amplification	3.1
MYC	MYC	Amplification	2.83 → 2.24
AR		Amplification	2.68
MYCN		Amplification	2.43
TP53	TP53	Deletion	1.67 → 1.88
CHEK2		Deletion	1.46
APC		Deletion	1.45
PTEN		Deletion	1.45
	AURKA	Amplification	3.01
	CCND3	Amplification	2.86
	EGFR	Amplification	2.20
	AKT3	Amplification	2.15
	PPP2R2A	Deletion	1.60
	NF2	Deletion	1.86
	FAT1	Deletion	1.88

Mutations that are detected in primary colon adenocarcinoma tissue biopsy based on a selected panel of genes are highlighted in bold.

## Discussion

Results from multiple studies in brain cancers, including LMD^11,12^ and diffuse gliomas,^1,4,5^ suggest that cfDNA in the CSF could serve as a liquid biopsy for diagnosing and/or monitoring these tumors. CSF is also of growing interest for the diagnosis of CNS lymphoma and could eventually supplant the need for biopsies in diseases that would otherwise not require neurosurgical intervention.^8,13^ CSF provides a valuable potential avenue to monitor CNS disease, particularly for LMD wherein tumor cells are in close contact with CSF.^3^ Importantly, tumor-derived cfDNA is not typically seen in parenchymal brain tumors without CSF contact, leading some to assume that detecting tumor-derived cfDNA indicates LMD. These cases demonstrate that metastatic tumor-derived cfDNA does not indicate LMD in patients whose tumors demonstrate ependymal contact. To our knowledge, 2 cases of metastatic brain tumors have previously been reported, which were in contact with CSF and resulted in positive cfDNA. However, this was only mentioned in a table footnote.^3^ We demonstrate that metastatic lesions with ventricular contact can also yield high levels of tumor cfDNA. As such, CSF cfDNA can be elevated in patients with metastatic tumors contacting a CSF surface, independent of LMD.

We used NGS, which enables a comprehensive analysis of tumor-associated alterations. Some testing services, such as MSK-IMPACT, require the submission of germline samples as a benchmark against tumor-derived cfDNA to confirm the tumor-associated origin of genetic alterations.^14^ In our case, this was not performed, leaving open the possibility that some identified mutations could have been of germline origin. Nevertheless, ample overlap between findings in tissue for pathognomonic tumor-associated alterations mitigated the need for germline samples in the presented cases.

In both patients, lumbar CSF was used at follow-up to help distinguish recurrent tumors from pseudo-progression despite the availability of a VP shunt. The reason was different in each case, but in both cases, sampling from a more distant lumbar location hampers direct comparison to the baseline ventricular data, where the highest cfDNA yield would be expected. For Patient 1, lumbar CSF was used partly for ease of access by a non-neurosurgical team and to help minimize the risk of inducing a procedure-related shunt infection. VP shunts are routinely accessed for diagnostic purposes and may be best for tumor monitoring. However, multidisciplinary coordination is critical to ensure sterile access by qualified personnel. Unlike Patient 1 who had widely open ventricles that would have been ideal to access via the VP shunt, the ventricles for Patient 2 were relatively narrow. The shunt catheter was also quite posterior in the ventricle, and CSF was not able to be readily accessed, as evidenced by limited recovery from the shunt (0.5 cc) prior to resection of the latent tumor. Importantly, cranial CSF has been reported to contain higher concentrations of cfDNA and other tumor biomarkers than lumbar CSF for diffuse gliomas.^15^ Fortunately, unlike tumor-associated oncometabolites like D-2-HG,^5^ the stability of cfDNA still enables lumbar CSF to be diagnostically useful.^15^ However, relative abundance cannot be meaningfully compared across sites.

Unlike CSF protein and metabolite biomarkers,^16^ each copy of tumor-associated cfDNA required a tumor cell to die for release. Tumor cell turnover is characteristic of growing tumors. The level of cfDNA release is not well characterized for previously treated latent tumors. Patient 2 had 0.0 ng/mL cfDNA after radiation despite a sizable residual tumor on MRI. This suggests that CSF cfDNA may not be optimal for the detection of inactive latent disease. Increased cfDNA from a low nadir may signal disease regrowth. However, optimally, quantitatively longitudinal monitoring would ideally serially sample CSF from the same location.

Plasma cfDNA is increasingly used for the detection and monitoring of systemic malignancies. In the case of CSF, cfDNA analysis has demonstrated higher sensitivity than cytology for diagnosing LMD^2,3^ and lymphoma.^13^ Circulating tumor DNA (ctDNA) analysis has shown potential for treatment stratification,^17^ and multiple groups are evaluating the utility of tumor-associated ctDNA in CSF. However, the field can only advance if CSF samples are regularly collected. The increasing clinical availability of CSF diagnostic assays may facilitate proactive, individualized longitudinal monitoring to help assess therapeutic response and disease trajectory.

At our institution, CSF banking through our institutional neuro-oncology biorepository is increasingly routine when CSF is accessed for clinical reasons. To help ensure relevant samples are available for ongoing discovery efforts, we have also initiated a CSF biomarkers protocol (NCT04692324), which enables VP shunt access and serial LPs for research in willing patients. Although the availability of a VP shunt provides a unique opportunity, the CNB analyses in Patient 2 illustrate the potential utility of even lumbar CSF, even when cfDNA abundance is relatively low.^4^ These efforts collectively enhance our ability to explore CSF-derived biomarkers and advance precision medicine in neuro-oncology.

## Conclusion

These 2 cases suggest the utility of NGS analysis of CSF cfDNA from parenchymal brain metastases abutting the ventricle. CSF cfDNA contained genetic alterations consistent with those identified in tumor tissue. These cases confirm that the utility of CSF cfDNA is not limited to patients with LMD. Further studies are needed to characterize the utility of CSF cfDNA for diagnosis, monitoring, and analysis of treatment response for patients with brain tumors in contact with CSF compartments.

## Data Availability

The data that support the findings of this study are available from the corresponding author upon reasonable request.
